# Longitudinal association of changes in diet with changes in body weight and waist circumference in subjects at high cardiovascular risk: the PREDIMED trial

**DOI:** 10.1186/s12966-019-0893-3

**Published:** 2019-12-27

**Authors:** Jadwiga Konieczna, Dora Romaguera, Veronica Pereira, Miguel Fiol, Cristina Razquin, Ramón Estruch, Eva M. Asensio, Nancy Babio, Montserrat Fitó, Enrique Gómez-Gracia, Emilio Ros, José Lapetra, Fernando Arós, Lluís Serra-Majem, Xavier Pintó, Estefanía Toledo, José V. Sorlí, Monica Bulló, Helmut Schröder, Miguel A. Martínez-González

**Affiliations:** 1 Research Group on Nutritional Epidemiology & Cardiovascular Physiopathology, Health Research Institute of the Balearic Islands (IdISBa), University Hospital Son Espases, Bldg I, Floor -1, Ctra de Valldemossa 79, 07120 Palma, Balearic Islands, Spain; 2 CIBER Fisiopatología de la Obesidad y Nutrición (CIBEROBN), Instituto de Salud Carlos III (ISCIII), Madrid, Spain; 3 Department of Preventive Medicine and Public Health, University of Navarra, C/ Irunlarrea, 1, 31080 Pamplona, Navarra Spain; 4 Department of Internal Medicine, IDIBAPS, Hospital Clinic, University of Barcelona, Barcelona, Spain; 5 Department of Preventive Medicine, University of Valencia, Valencia, Spain; 6 Universitat Rovira i Virgil. Departament de Bioquímica i Biotecnologia. Unitat de Nutrició Humana, IISPV, University Hospital of Sant Joan de Reus, Reus, Spain; 7 Lipids and Cardiovascular Epidemiology Research Unit, Institut Municipal d’Investigació Mèdica (IMIM), Barcelona, Spain; 8 Department of Preventive Medicine, University of Malaga, Malaga, Spain; 9 Lipid Clinic, Department of Endocrinology and Nutrition, Institut d’Investigacions Biomèdiques August Pi Sunyer (IDIBAPS), Hospital Clínic, Barcelona, Spain; 10 Department of Familiy Medicine, Primary Care Division of Sevilla, San Pablo Health Center, Sevilla, Spain; 11 Department of Cardiology, University Hospital of Alava, Vitoria, Spain; 12 University of Las Palmas de Gran Canaria, Research Institute of Biomedical and Health Sciences (IUIBS), Preventive Medicine Service, Centro Hospitalario Universitario Insular Materno Infantil (CHUIMI), Canarian Health Service, Las Palmas, Spain; 13 Lipids and Vascular Risk Unit, Internal Medicine, Hospital Universitario de Bellvitge-IDIBELL, Hospitalet de Llobregat, Barcelona, Spain; 14 CIBER de Epidemiología y Salud Pública (CIBERESP), Instituto de Salud Carlos III, Madrid, Spain; 15 Cardiovascular Risk and Nutrition Research Group (CARIN), Institut Hospital del Mar d’ Investigacions Mèdiques (IMIM), Barcelona Biomedical Research Park, Barcelona, Spain; 16 Department of Nutrition, Harvard T. H. Chan School of Public Health, Boston, MA USA

**Keywords:** Dietary intake, Body weight, Waist circumference, Longitudinal study, repeated-measures data, The PREDIMED trial

## Abstract

**Background:**

Consumption of certain foods is associated with long-term weight gains and abdominal fat accumulation in healthy, middle-aged and young, non-obese participants. Whether the same foods might be associated with changes in adiposity in elderly population at high cardiovascular risk is less known.

**Objective:**

Using yearly repeated measurements of both food habits and adiposity parameters, we aimed to investigate how changes in the consumption of specific foods were associated with concurrent changes in weight or waist circumference (WC) in the PREDIMED trial.

**Design:**

We followed-up 7009 participants aged 55–70 years at high cardiovascular risk for a median time of 4.8 years. A validated 137-item semi-quantitative Food Frequency Questionnaire was used for dietary assessment with yearly repeated measurements. We longitudinally assessed associations between yearly changes in food consumption (serving/d) and concurrent changes in weight (kg) or WC (cm).

**Results:**

Yearly increments in weight were observed with increased consumption (kg per each additional increase in 1 serving/d) for refined grains (0.32 kg/serving/d), red meat (0.24), potatoes (0.23), alcoholic beverages (0.18), processed meat (0.15), white bread (0.07) and sweets (0.04); whereas inverse associations were detected for increased consumption of low-fat yogurt (− 0.18), and low-fat milk (− 0.06).

Annual WC gain (cm per each additional increase in 1 serving/d) occurred with increased consumption of snacks, fast-foods and pre-prepared dishes (0.28), processed meat (0.18), alcoholic beverages (0.13), and sweets (0.08); whereas increased consumption of vegetables (− 0.23), and nuts (− 0.17), were associated with reductions in WC.

**Conclusions:**

In this assessment conducted in high-risk subjects using yearly repeated measurements of food habits and adiposity, some ultra-processed foods, refined carbohydrates (including white bread), potatoes, red meats and alcohol were associated with higher weight and WC gain, whereas increases in consumption of low-fat dairy products and plant foods were associated with less gain in weight and WC.

**Trial registration:**

This study was registered at controlled-trials.com with International Standard Randomized Controlled Trial Number (ISRCTN): 35739639. Registration date: 5 October 2005.

## Introduction

Findings on the relationships between dietary patterns with beneficial or deleterious effects on health are well established and constitute the basis for existing dietary guidelines. However, further food- and nutrient-based research is warranted to elucidate the mechanisms by which dietary patterns exert their effects. Identification the most likely causative foods would strengthen the evidence on which dietary recommendations for preventing obesity and weight gain based on healthy dietary patterns can be developed [1, 2].

Food groups have been addressed in a prospective large and long-term study from three American cohorts, which found that increments in the consumption of potato chips, potatoes, sugar-sweetened beverages (SSB), as well as red and processed meat was linked to higher weight gain, whereas the intake of vegetables, fruits, nuts, whole grain, and yogurt was linked to lower weight gain [3, 4]. A similar study conducted in Europeans, found that a diet high in fruit and dairy products and low in bread, processed meat, margarine, and soft drinks was associated with lower abdominal fat accumulation over time [5]. However, in both cohorts only middle-aged and young individuals (all < 65 years) with no chronic disease, and no obesity (Americans) were evaluated.

Prospective studies on diet and obesity typically evaluated only prevalent, baseline food intake [5] and subsequent changes in weight, or the association between dietary changes within a period of several years and weight changes occurred thereafter. As eating behaviors change over time, Smith et al proposed that the optimal methodology consists on the analysis of changes in diet with concurrent changes in weight, both evaluated along the same timespan [6]. In addition, previous studies [3, 4] only included dietary measurements repeated every 4 years, but none of them repeatedly assessed diet every year. As the induction period for the association between diet and weight gain is likely to be shorter than 4 years, yearly assessments of diet and weight can provide further insights into causally relevant associations.

The PREDIMED trial evaluated the effect of Mediterranean diet (MedDiet) *vs* a low-fat diet, on cardiovascular disease (CVD) prevention in a large sample of elderly subjects at high CVD risk. Over the course of the trial, all participants experienced small weight losses but (also small) waist circumference (WC) increments; this could be related to the age-related decline in lean body mass and fat mass redistribution, leading to visceral fat accumulation. For this reason, WC might be a better measure of adiposity in elderly populations than other anthropometric measurements such as body weight or body mass index (BMI); this is also supported by the fact that the association between overweight measured using the BMI and mortality is not yet well established in the elderly population [7–9]. In the PREDIMED study, those exposed to MedDiet lost slightly more weight and gained less in WC than subjects on control diet [10].

The PREDIMED is unique in having conducted repeated dietary assessments with the same 137-item semi-quantitative Food Frequency Questionnaire (FFQ) at baseline and on a yearly basis thereafter. In addition, the PREDIMED study consists of a population of elderly participants at high cardiovascular risk, for which the evidence on how diet might influence differently overall *vs* central adiposity is not yet well established. Using these FFQs, we aimed to investigate, in an elderly population at high CVD risk, how yearly changes in dietary factors were simultaneously associated with weight and WC changes measured directly and objectively during each of the 5 first years of follow-up.

## Subjects and methods

### Study overview and participants

The current study is based on data from the PREDIMED (Prevención con Dieta Mediterránea) trial (ISRCTN35739639) over the first 5 years of follow-up. Details of the trial have been previously published [11, 12]. Briefly, PREDIMED was a randomized, controlled trial started in 2003 in 11 Spanish centers (www.predimed.es), and designed to compare the effect of interventions with MedDiet supplemented with extra-virgin olive oil (EVOO) or nuts *vs* a control low-fat diet, on primary prevention of CVD. Study population included men and women aged 55–80 years, free of CVD at enrolment, but at high CVD risk due to the presence of either type 2 diabetes (T2D) or at least three major risk factors (hypercholesterolemia, low high-density lipoprotein, overweight/obesity, hypertension, current smoking or family history of premature coronary heart disease). The trial was stopped in December 2010 (median follow-up of 4.8 years), when the evidence of early cardiovascular benefit of MedDiet groups *vs* control group became identified. All participants provided written informed consent and the study protocol was approved by the institutional review boards of all recruiting centers according to the Declaration of Helsinki. This study followed the STROBE-nut guidelines for reporting [13].

Of the total 7447 participants included in the PREDIMED trial, 291 participants were excluded due to the missing data on diet, anthropometrics or other covariables at baseline, and 147 participants due to total energy intake values outside predefined limits (500–3500 kcal/d (women), 800–4000 kcal/d (men)) [14], resulting in a final number of 7009 participants (see the flow chart in Additional file 1: Figure S1).

### Assessment of changes in diet

Data on dietary intake over the last year was assessed with the use of a validated 137-item semi-quantitative FFQ administered by trained dietitians at baseline and yearly during follow-up in a face-to-face interview [15]. Intake of each food item was calculated by multiplying serving size by frequency of consumption (from never to > 6 times/d). Spanish food composition tables were used to derive total energy (kcal/d) and nutrient intake [16, 17]. Food items have been grouped if they shared similarities in nutritional characteristics, and plausibly, were likely to exert similar biologic effects on obesity risk. The exposures of interest were changes in the intake of 31 specific food groups expressed in serving/d, calculated as the difference between yearly measured values and values from the previous year. Detailed information on the specific food groups, and items used to assess dietary change is available in Table 1 and Additional file 1: Table S1.

**Table 1:** Socio-demographic, lifestyle and dietary characteristics of participants at baseline, during follow-up and average change over 5-year of follow-up

Parameters	Baseline Mean (SD)	Average during follow-up Mean (SD)	Average yearly change during follow-up Mean (5th, 95th percentiles)
Socio-demographic and lifestyle			
Males sex (%)	42		
Age (years)	67.0 (6.2)		
Body weight (kg)	76.7 (12.0)	76.4 (12.1)	−0.13 (−2.89, 2.60)
BMI (kg/m^2^)	30.0 (3.8)	29.9 (3.9)	−0.05 (− 1.15, 1.00)
Obesity prevalence (%)	47		
Waist circumference (cm)	100 (10)	100 (10)	−0.04 (−5.00, 5.00)
Abdominal obesity prevalence (%)	73		
Diabetes prevalence (%)	48		
Current smoker (%)	14		
Higher education/technician (%)	7		
Physical activity (METs. min/d)	233 (239)	250 (213)	3.96 (− 155, 163)
Total energy intake (kcal/d)	2239 (543)	2189 (432)	−33.4 (− 487, 429)
Adherence to MedDiet (14-points score)	9 (2)	10 (2)	0.5 (−1.0, 2.5)
Food groups intake (serving/d)			
EVOO	2.11 (2.31)	3.48 (2.13)	0.49 (−1.50, 2.50)
Nuts	0.34 (0.46)	0.57 (0.54)	0.07 (−0.36, 0.65)
Fruits	1.84 (1.01)	1.98 (0.78)	0.05 (−0.75, 0.93)
Low-fat milk	1.07 (0.99)	1.11 (0.81)	0.04 (−0.75, 0.83)
Fish and seafood	0.87 (0.44)	0.92 (0.32)	0.01 (−0.33, 0.36)
Low-fat yogurt	0.29 (0.41)	0.32 (0.36)	0.01 (−0.31, 0.32)
White meat	0.44 (0.26)	0.47 (0.20)	0.01 (−0.22, 0.26)
Legumes	0.14 (0.08)	0.15 (0.06)	0.00 (−0.06, 0.07)
Vegetables	1.51 (0.68)	1.53 (0.53)	0.00 (−0.50, 0.54)
Eggs	0.33 (0.19)	0.34 (0.14)	0.00 (−0.14, 0.14)
Refined grains	0.34 (0.24)	0.33 (0.17)	0.00 (−0.18, 0.21)
Breakfast cereals	0.10 (0.35)	0.11 (0.30)	0.00 (−0.14, 0.19)
Whole bread	0.37 (0.78)	0.35 (0.57)	−0.01 (− 0.57, 0.50)
Snacks, fast foods and pre-pared dishes	0.32 (0.41)	0.31 (0.34)	−0.01 (− 0.34, 0.28)
SSB	0.18 (0.43)	0.15 (0.29)	−0.01 (− 0.29, 0.22)
Natural juices	0.14 (0.33)	0.13 (0.25)	−0.01 (− 0.22, 0.22)
ASB	0.11 (0.42)	0.09 (0.31)	−0.01 (− 0.20, 0.14)
Margarine	0.08 (0.26)	0.06 (0.17)	−0.01 (− 0.17, 0.09)
Butter	0.04 (0.18)	0.03 (0.13)	−0.01 (− 0.07, 0.03)
Cheese	0.59 (0.52)	0.54 (0.37)	−0.02 (− 0.43, 0.36)
Potatoes	0.54 (0.32)	0.51 (0.21)	−0.02 (− 0.36, 0.28)
Whole-fat yogurt	0.12 (0.29)	0.09 (0.19)	−0.02 (− 0.25, 0.14)
Whole-fat milk	0.23 (0.60)	0.15 (0.43)	−0.03 (− 0.39, 0.09)
Alcoholic beverages	0.78 (1.29)	0.71 (1.06)	−0.03 (− 0.60, 0.50)
Red meat	0.39 (0.29)	0.30 (0.20)	−0.04 (− 0.27, 0.16)
White bread	1.24 (1.19)	1.16 (0.90)	−0.04 (−1.00, 0.86)
Coffee/tea	1.46 (1.10)	1.35 (0.87)	−0.04 (− 0.91, 0.79)
Processed meat	0.87 (0.66)	0.71 (0.41)	−0.06 (− 0.59, 0.38)
Other vegetable oils	0.30 (0.83)	0.10 (0.36)	−0.09 (− 0.50, 0.07)
Sweets	2.34 (2.68)	1.94 (2.10)	−0.19 (−2.22, 1.37)
Olive oil	1.75 (2.00)	0.97 (1.42)	−0.26 (−2.50, 1.25)

Values are mean (SD) for baseline and follow-up continuous variables, and percentages for categorical variables. Average changes during follow-up were assessed as means with censoring of data for interquartile range (5th, 95th percentile). Obesity was defined as BMI ≥30 kg/m^2^, and abdominal obesity as waist circumference ≥ 88 cm (women) and ≥ 102 cm (men). *MedDiet* Mediterranean Diet (14-points score), *ASB* artificially-sweetened beverages, *BMI* body mass index, *EVOO* extra virgin olive oil, *SSB* sugar-sweetened beverages

### Assessment of changes in anthropometry

At baseline and at each annual visit body weight, WC (determined midway between the lowest rib and the iliac crest), and height were precisely measured in duplicate (the average of these 2 measurements was the analyzed value) by dietitians trained and hired for the trial. Dietitians used calibrated scales, anthropometric tape and a wall-mounted stadiometer, respectively; and body mass index (BMI) was calculated. Absolute changes in body weight and WC were calculated as the difference between yearly measured values and values from the previous year.

### Assessment of other covariables

A general medical questionnaire was administered on a yearly basis to obtain information on age, sex, educational level, smoking status, hormonal replacement therapy in case of women, T2D status (medically-diagnosed condition self-reported by the participant at inclusion). Leisure-time total physical activity (METs. min/d) was assessed with the Minnesota Leisure-Time Physical Activity Questionnaire validated previously in Spanish men and women [18, 19].

### Statistical analyses

Characteristics of the study participants at baseline and during follow-up are presented as mean (SD) for continuous and percent for categorical variables. The average yearly change in each characteristic is presented as mean (5th, 95th percentile).

Multivariable generalized estimating equation (GEE) analyses with robust standard error and autoregressive correlations (based on observed correlation matrices), were used to estimate associations between yearly changes in the consumption of each of the 31 food groups (serving/d) yearly changes in weight (kg) and WC (cm) (all continuous variables). All the models were adjusted for the minimally sufficient adjustment set of covariables, determined using Directed Acyclic Graph (DAG) [20], as implemented in DAGitty software (www.dagitty.net) [21] (Additional file 1: Figure S2): time (years, because there were slight variations in the period between successive visits), sex, center, intervention group (combined MedDiets or control group), age, baseline BMI/WC (depending on outcome) and educational level (higher education/technician, secondary education, primary education/illiterate or missing data), as well as yearly measured changes in smoking status (never, current or former) and physical activity (METs. min/d).

Moreover, for food groups for which the associations with changes in anthropometry were statistically significant, the models were additionally adjusted for intake of other foods simultaneously, to estimate mutually adjusted associations. No multi-collinearity was observed between these food groups (tolerance 0.963–0.996).

The principal analyses were conducted by coding and imputing missing data on changes in body weight, WC, and diet during follow-up with carried-forward values (LOCF) up to last follow-up, death, or the date on which the participant was lost to follow-up, whichever occurred first.

In order to summarize the observed associations between dietary exposures and anthropometry, two global dietary scores (GDSs) were estimated separately for body weight (GDS-Wt) and WC (GDS-WC). For this purpose, yearly changes in the consumption of each food group, which were found to be independently associated with changes in each of our outcome variables were classified into quintiles (Additional file 1: Table S2). For each quintile, different values were assigned ranging from 1 to 5 for these food groups that were inversely associated, or 5 to 1 for foods associated positively with changes in our outcomes. The final score was created summing all of these values, which could vary from 9 to 45 points for body weight and from 6 to 30 points for WC; a higher score indicated a dietary pattern inversely associated with weight gain or WC gain. This procedure was repeated for each yearly repeated measurement of diet. To allow direct comparison across scores, GDSs were normalized into z-scores.

In order to minimize false discovery rate due to multiple comparisons, GDSs were used as a summary measure of the overall exposure to a dietary pattern associated with anthropometry, to perform further sensitivity analyses. In that way, plausible interactions between foods within each score were taken into account, capturing their potential synergistic effects. Subgroup analyses were conducted to explore consistency of the associations found across selected subsets according to sex, age at enrollment (< 65 or ≥ 65 years), baseline overall obesity (BMI < 30 or ≥ 30 kg/m^2^), abdominal obesity (WC < 88 (women) and < 102 cm (men) or ≥ 88 (women) and ≥ 102 cm (men)) and diabetes status (yes/no), as well as intervention group (combined MedDiets or control diet). Analysis across intervention groups was also adjusted for propensity scores (built with 30 baseline covariables) to control for minor (not clinically significant) imbalances in baseline covariables. Tests for GDSs by stratum interactions were also performed.

As part of the sensitivity analyses, analyses with use of GDSs, as well as with the food groups that were found to be significantly associated with outcomes, were repeated considering observed data (without LOCF imputation), additionally adjusting for changes in total energy intake (to mitigate the effects of measurement error in collected data using self-reported FFQ), and excluding those patients who died during the follow-up period from any cause (5% participants). Moreover, as reported elsewhere [12], in further sensitivity analyses, we took into account deviations from the randomization protocol, excluding 2nd household members and one small clinic (Site D), where participants were not individually allocated (13% participants). Finally, in order to control for overall healthy dietary pattern associated with our food groups, the multivariable model was additionally adjusted for yearly measured changes in adherence to Mediterranean Diet (MedDiet), assessed with a validated in adult Spanish population 14-item Mediterranean Diet Adherence Screener [22].

Statistical analyses were performed using Stata v15.0 program, with statistical significances set at *p* < 0.05.

## Results

In the overall cohort of PREDIMED (Table 1), the greatest changes in dietary habits were observed for increased consumption of EVOO, nuts, and fruits, as well as for decreased consumption of olive oil, sweets and other vegetable oils. These changes are likely to be partly explained by the effects of the dietary intervention. Changes in weight and WC during follow-up were of small magnitude.

**Fig. 1:**
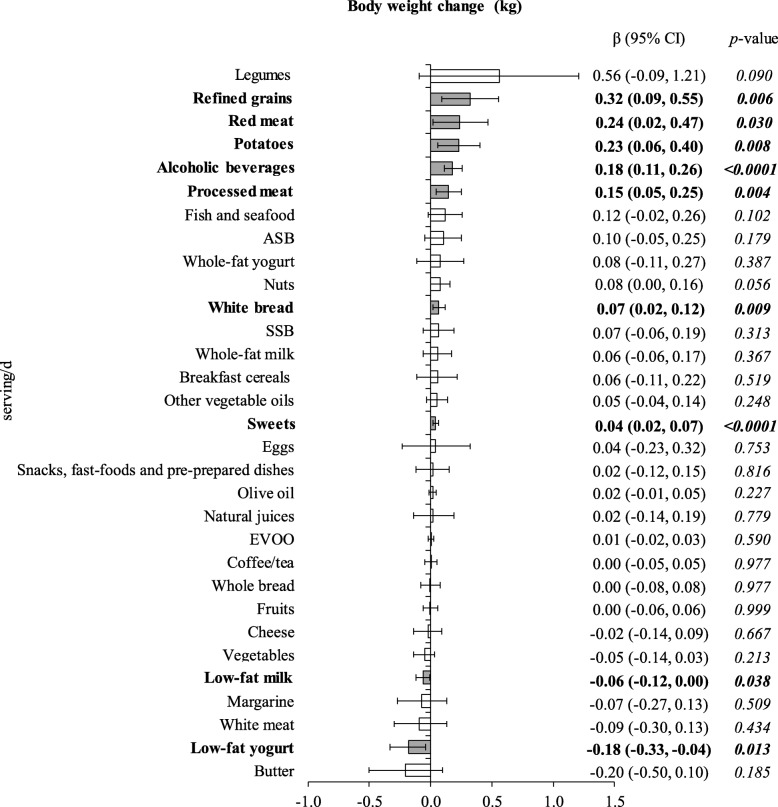
Association of yearly changes in food groups intake with concurrent changes in body weight over 5-year of follow-up. β (95% CI) represents the yearly change in body weight (kg) associated with increased/decreased consumption of particular food groups (serving/d). Models used for generalized estimating equation analysis were run separately for each food group and were adjusted for time, sex, center, intervention group, age, baseline BMI and educational level, as well as yearly measured changes in smoking status and physical activity. ASB – artificially-sweetened beverages; EVOO – extra virgin olive oil; SSB – sugar-sweetened beverages

**Fig. 2:**
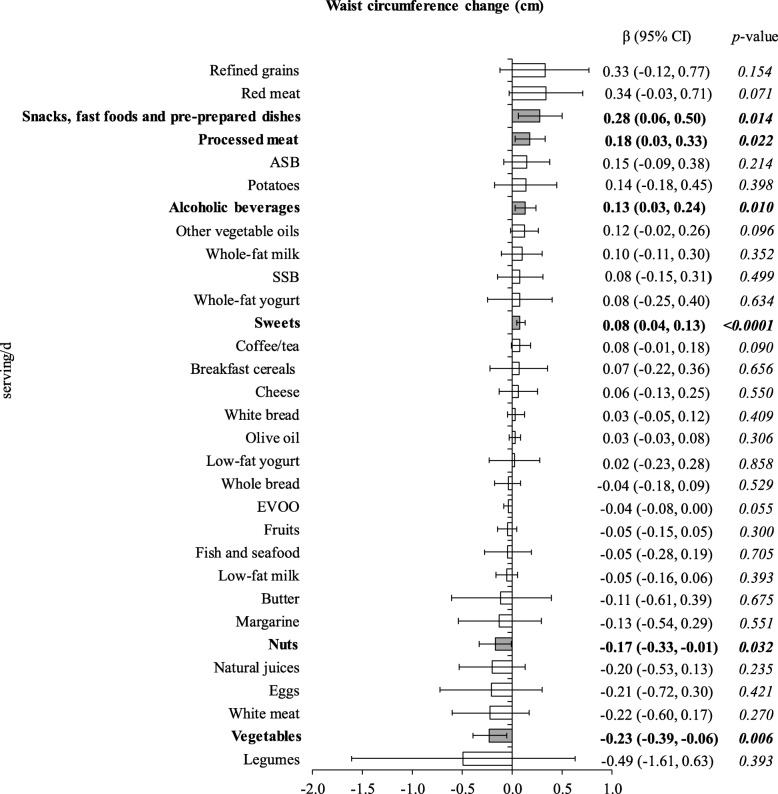
Association of yearly changes in food groups intake with concurrent changes in waist circumference over 5-year of follow-up. β (95% CI) represents the yearly change in waist circumference (cm) associated with increased/decreased consumption of particular food groups (serving/d). Models used for generalized estimating equation analysis were run separately for each food group and were adjusted for time, sex, center, intervention group, age, baseline waist circumference and educational level, as well as yearly measured changes in smoking status and physical activity. ASB – artificially-sweetened beverages; EVOO – extra virgin olive oil; SSB – sugar-sweetened beverages

The associations between yearly changes in food group consumption and concurrent body weight and WC changes are shown in Figs. 1 and 2, respectively. Significant increments in weight were observed with increased consumption of refined grains (β 0.32 kg per each increment of 1 additional serving/d; 95% CI 0.09, 0.55 kg), red meat (0.24; 0.02, 0.47), potatoes (0.23; 0.06, 0.40), alcoholic beverages (0.18; 0.11, 0.26), processed meat (0.15; 0.05, 0.25), white bread (0.07; 0.02, 0.12), and sweets (0.04; 0.02, 0.07); whereas lower weight gain was observed with increments in consumption of low-fat yogurt (− 0.18; − 0.33, − 0.04), and low-fat milk (− 0.06; − 0.12, 0.00) (Fig. 1). In turn WC gain occurred with yearly increases in the consumption of snacks, fast-foods and pre-prepared dishes (0.28 cm per each additional increase in 1 serving/d; 0.06, 0.50 cm), processed meat (0.18; 0.03, 0.33), alcoholic beverages (0.13; 0.03, 0.24), and sweets (0.08; 0.04, 0.13); whereas yearly increments in consumption of vegetables (− 0.23; − 0.39, − 0.06) and nuts (− 0.17; − 0.33, − 0.01) were associated with less WC gains (Fig. 2).

Mutual adjustment for food groups significantly associated with our outcomes revealed that the overall magnitude and direction of associations shown in Figs. 1 and 2 persisted, showing that the associations of these food groups with anthropometric changes were independent from each other (Table 2). Only the association between increments in consumption of potatoes (0.16 kg; − 0.01, 0.33 kg; *p* = 0.071), refined grains (0.21; − 0.02, 0.44; *p* = 0.075), processed meats (0.10; − 0.01, 0.20; *p* = 0.065) and red meats (0.14; − 0.08, 0.37; *p* = 0.213) and weight gain were somewhat attenuated (Table 2a).

**Table 2:** Sensitivity analysis: association of yearly changes in food groups intake with concurrent changes in body weight (A) or waist circumference (B) over 5-year of follow-up - mutual adjustment considering food groups significantly associated with changes in anthropometry

Food group (serving/d)	β (95% CI)	*p*-value
A. Body weight change (kg)		
Refined grains	0.21 (− 0.02, 0.44)	0.075
Alcoholic beverages	0.17 (0.09, 0.24)	< 0.0001
Potatoes	0.16 (−0.01, 0.33)	0.071
Red meat	0.14 (−0.08, 0.37)	0.213
Processed meat	0.10 (−0.01, 0.20)	0.065
White bread	0.05 (0.00, 0.10)	0.059
Sweets	0.04 (0.01, 0.06)	0.005
Low-fat milk	−0.07 (−0.13, − 0.01)	0.031
Low-fat yogurt	−0.15 (− 0.30, − 0.01)	0.035
B. Waist circumference change (cm)		
Snacks, fast foods and pre-pared dishes	0.24 (0.02, 0.46)	0.032
Processed meat	0.15 (0.00, 0.31)	0.054
Alcoholic beverages	0.12 (0.02, 0.22)	0.023
Sweets	0.08 (0.03, 0.12)	< 0.0001
Nuts	− 0.16 (− 0.32, 0.00)	0.052
Vegetables	− 0.24 (− 0.41, − 0.08)	0.004

β (95% CI) represents the yearly change in body weight (kg) or waist circumference (cm) associated with increased/decreased consumption of particular food groups (serving/d). The mutually adjusted model was adjusted for covariables used in all multivariable models (time, sex, center, intervention group, age, baseline BMI/WC (depending on outcome) and educational level, as well as yearly measured changes in smoking status and physical activity) and for the other food groups significantly associated with changes in anthropometry

Then, food groups that showed significant association with each of anthropometry variables were analyzed in combination using a GDS. Overall, yearly increments in each GDS (per 1 SD) were associated with yearly weight changes of − 0.16 kg (95% CI -0.21, − 0.12 kg; *p* < 0.0001) and WC changes of − 0.27 cm (− 0.35, − 0.20 cm; *p* < 0.0001) (Table 3). Furthermore, in subgroup analyses we found that the inverse association between diet change and concurrent weight change was stronger among non-diabetics (β − 0.25 kg; 95% CI -0.32, − 0.18 kg; *p* < 0.0001) than among diabetics (− 0.07 kg; − 0.13, − 0.00 kg; *p* = 0.037) (*p* for interaction 0.0001). Moreover, we found that the association between GDS-WC and WC was more pronounced in women (− 0.35 cm; − 0.46, − 0.23 cm; *p* < 0.0001) than in men (− 0.18 cm; − 0.28, − 0.09 cm; *p* < 0.0001) (*p* for interaction 0.019), which could be due to the fact that in women more food groups were significantly associated with changes in WC (Additional file 1: Table S3). Finally, association between GDS-WC and WC was higher in abdominally obese participants (− 0.31 cm; − 0.41, − 0.22 cm; *p* < 0.0001) than in non-abdominally obese (− 0.18 cm; − 0.31, − 0.05 cm; *p* = 0.007) (*p* for interaction 0.042) (Table 3).

**Table 3:** Association of yearly changes in global dietary scores (GDSs) with concurrent changes in body weight or waist circumference over 5-year of follow-up by subgroups

*n* = 7009	Body weight change (kg)		Waist circumference change (cm)	
	β (95% CI)	*p*-value	β (95% CI)	*p*-value
GDS (per 1 SD)	−0.16 (− 0.21, − 0.12)	< 0.0001	−0.27 (− 0.35, − 0.20)	< 0.0001
Sex				
Men (*n* = 2986)	− 0.14 (− 0.21, − 0.07)	< 0.0001	−0.18 (− 0.28, − 0.09)	< 0.0001
Women (4023)	−0.18 (− 0.25, − 0.12)	< 0.0001	−0.35 (− 0.46, − 0.23)	< 0.0001
*p* for interaction		*0.498*		*0.019*
Age				
< 65 (*n* = 2688)	−0.19 (− 0.26, − 0.11)	< 0.0001	−0.24 (− 0.36, − 0.13)	< 0.0001
≥ 65 (*n* = 4321)	−0.15 (− 0.21, − 0.09)	< 0.0001	−0.29 (− 0.39, − 0.19)	< 0.0001
*p* for interaction		*0.371*		*0.591*
Obesity status				
Non-obese (*n* = 3723)	−0.15 (− 0.21, − 0.09)	< 0.0001	−0.21 (− 0.32, − 0.11)	< 0.0001
Obese (*n* = 3286)	−0.19 (− 0.26, − 0.11)	< 0.0001	−0.35 (− 0.47, − 0.24)	< 0.0001
*p* for interaction		*0.445*		*0.076*
Abdominal obesity status				
Non-abdominal obese (*n* = 1881)	−0.10 (− 0.18, − 0.02)	0.016	−0.18 (− 0.31, − 0.05)	0.007
Abdominal obese (*n* = 5128)	−0.19 (− 0.25, − 0.13)	< 0.0001	−0.31 (− 0.41, − 0.22)	< 0.0001
*p* for interaction		*0.107*		*0.042*
Diabetes status				
Non-diabetics (*n* = 3618)	−0.25 (− 0.32, − 0.18)	< 0.0001	−0.27 (− 0.38, − 0.17)	< 0.0001
Diabetics (*n* = 3391)	−0.07 (− 0.13, 0.00)	0.037	−0.27 (− 0.38, − 0.16)	< 0.0001
*p* for interaction		*0.0001*		*0.864*
Intervention group				
Controls (*n* = 2298)	−0.19 (− 0.27, − 0.11)	< 0.0001	−0.33 (− 0.48, − 0.18)	< 0.0001
Intervention (*n* = 4711)	−0.15 (− 0.21, − 0.10)	< 0.0001	−0.25 (− 0.34, − 0.17)	< 0.0001
*p* for interaction		*0.317*		*0.503*

β (95% CI) represents difference in yearly change in body weight (kg) or waist circumference (cm) associated with increments of each GDS (per 1 SD). Model used for generalized estimating equation analysis was adjusted for time, sex, center, intervention group, age, baseline BMI/WC (depending on the outcome) and educational level, as well as yearly measured changes in smoking status and physical activity. Obesity was defined as BMI ≥30 kg/m^2^, and abdominal obesity as waist circumference ≥ 88 cm (women) and ≥ 102 cm (men)

The associations between GDS and anthropometry did not change after conducting additional sensitivity analyses (Additional file 1: Table S4).

No relevant changes in effect estimates were detected in the sensitivity analyses conducted for food groups that were significantly associated with weight and WC changes (Additional file 1: Table S5); only, the association between consumption of red meat, processed meat, potatoes, bread and refined grains with weight changes was attenuated after adjusting for concurrent changes in energy intake. On the contrary, associations with WC changes were not modified after adjusting for energy.

## Discussion

In our long-term assessment, in an elderly population at high CVD risk, we found that an increments in the consumption of some ultra-processed foods such as snacks, fast-foods, pre-prepared dishes, processed meat, and sweets was associated with changes in WC; whereas increased consumptions of high glycemic index (GI) foods (including white bread, refined grains, potatoes, and sweets) and red and processed meats were associated solely with weight gain. Alcohol intake was associated with higher gains in both weight and WC. In turn, increased intake of some low-fat dairy products was associated with less weight, and intake of vegetables and nuts with less WC gain. In case of the associations between some food items (i.e. potatoes, red and processed meats) and weight change, these associations were attenuated after mutual adjustment for each other or after adjustment for energy; in contrast, most associations with WC changes remained significant after these adjustments. Importantly, these findings were obtained after using repeated yearly measurements of concurrent changes in foods and anthropometry, both evaluated along the same timespan. This is a novelty because previous studies using repeated measurements of diet and weight to assess concurrent changes considered a wider timespan (4 years instead of 1 year) and they relied only in self-reported measures of weight, but not in objectively measured weight and waist [3, 4]. In the PREDIMED trial anthropometric variables were repeatedly collected in duplicate (the average of 2 measurements was the analyzed value) every year by dietitians, specifically trained to follow the measurement protocol of the trial.

This work emphasizes previous findings on importance of carbohydrate quality for obesity prevention [23, 24]. Foods that are low in fiber but high in refined carbohydrates or starches, with a high GI, i.e. white bread, refined grains, potatoes or sweets, were associated with higher weight gains. These results are consistent with previous longitudinal studies [3, 25, 26] and short-term trials [27]. Thus, it is likely that the observed positive link between refined carbohydrates or starches and weight gain is mediated through nutritional factors, such as fiber content, GI and added simple sugars. High-GI foods are less satiating, inducing hunger and overconsumption, and enhance lipogenesis, as compared to low-GI and high-fiber foods [27, 28]. Nevertheless, mutual adjustment for other food groups attenuated the link between refined grains and potatoes with weight gain, suggesting that this association may be explained by combining effect of other unfavorable foods usually consumed with refined grains and potatoes i.e. red and processed meat. Similarly, after adjusting for changes in energy, these associations were attenuated, which may indicate that a plausible mechanism of action is related to alterations of the energy balance.

Low-fat dairy products have been included in American Dietary Guidelines for disease prevention and overall health. In line with these recommendations we found an inverse association between the intake of both low-fat milk and low-fat yogurt and changes in weight; while no association was found for whole-fat milk and yogurt. The plausible mechanism might be related to calcium, casein or biopeptides, whether it could be related to fat content deserves future investigations. For comparison, prior longitudinal studies evaluating dairy-obesity relationships showed protective roles for high-fat dairy products against weight gain, and reported null association for low-fat dairy in younger and healthier American cohorts [29, 30]. The discrepancy between the findings across longitudinal studies might be attributed to differences in exposure variable categorization and measurement (baseline intake or changes over time); or to the fact that whole-fat dairy is not consumed much in this cohort, potentially limiting the ability to detect possible associations.

In concordance with a majority of prior prospective reports [3, 4, 31], this study supports the hypothesis that red and processed meat predict long-term weight gains. This positive association might be attributed to high-energy density, high content of cholesterol, saturated fatty acid (SFA), sodium and other additives as nitrates (processed meat), as well as synergistic effect with detrimental dietary or lifestyle patterns associated with meat intake [26, 32]. It has been also postulated that intake of dietary protein may help to maintain lean mass in older adults [33], thus the use of body weight as outcome rather than adiposity might be misleading. In our previous analysis conducted in other cohort of older subjects with overweight/obesity and metabolic syndrome, we have postulated that alternative measures of WC might serve as plausible options to assess changes in adiposity, in case in which more precise methods cannot be applied. In this regard, our investigation found positive associations between processed meat and red meat (the latter statistically non-significant), with WC changes. This is in line with the results from a recent systematic review and meta-analysis of observational studies [32].

Regarding alcohol use, prior studies reported a positive association with weight gain among heavy drinkers and spirits drinkers, whereas light-to-moderate alcohol intake, especially wine, was found protective [34]. In our investigation, overall alcohol intake was consistently and positively associated with weight and WC gain, as well as in two other large longitudinal studies among middle-aged Europeans and Americans [3, 5]. Our secondary analysis showed that the different alcohol subtypes showed similar associations with weight and waist. Despite high energy content, the plausible mechanisms may include appetite enhancement [35] and lipid oxidation reduction [36], but also consumption of alcohol might mirror unhealthy lifestyles that lead to overall and abdominal obesity. However, futures studies with comprehensive set of confounders and modifiers (frequency, subtypes, alcohol history and obesity tendency) are warranted.

Recently, an increased attention has been given to the detrimental association between SSB consumption and obesity or weight gain [37]. In turn, in our analysis we could not support those findings. However, the point estimates and most of the confidence interval showed results that were compatible with increased weight and WC in association with SSB consumption. The wide confidence intervals could be attributed to the fact that previous studies included participants of younger age groups [3, 5], in which the consumption of SSB is usually higher than that observed in the PREDIMED cohort.

Convenient and palatable ultra-processed foods contribute to obesity epidemic, supplying excessive amount of SFA, trans fat, added sugars, and sodium [38, 39]. In this regard, in our study WC gain was associated with higher consumption of sweets, snacks, fast-foods and pre-prepared dishes. Similarly, prior large longitudinal studies of younger US and European citizens found that fast foods, French fries, potato chips, sweets were associated with higher WC [5, 40]. Thus, abdominal obesity prevention should put particular attention on these foods, which due to its hyper-palatability and conveniency tend to be consumed in excess replacing healthier and more satiating options.

Dietary fiber has been associated with less visceral fat [41], and the possible mechanisms include satiety effect released by increasing food volume and lowering glycemic and insulinemic response to a meal [42], as well as improvement of gut microbiome. An inverse association of vegetables intake with WC changes in our study is in accordance with previous longitudinal studies using baseline dietary information [5, 40]. However, unlike in those studies, we did not reveal an inverse association with fruits; this might be attributed to the seasonal fluctuations in fruits intake and warrants future studies. Furthermore, nuts are a special case of fiber-rich food that is also rich in fats, albeit mostly unsaturated. It is likely that despite fiber and bioactive compounds, vegetable protein and fatty acids found in nuts exert anti-obesity effect by increasing thermogenesis, resting energy expenditure and oxidation [43, 44]. Previous analysis based on the present cohort showed that an increase in vegetable fat intake from natural sources as nuts and EVOO, implemented in the setting of MedDiet, has significant effect on weight reduction and lesser age-related increases in WC [10]. In this longitudinal analysis we found nuts negatively associated with changes in WC, but not weight. Future investigations should establish whether the association on abdominal obesity is due to specific nutrient properties or displacement of unfavorable foods.

We acknowledge several study limitations, as these analyses are exploratory within the PREDIMED trial, and findings are limited to white, elderly Spanish subjects at high CVD risk. Whilst changes in adiposity were objectively measured at each time point (WC and body weight), anthropometry is less accurate than direct methods (i.e. imaging techniques) to assess changes in body composition in elderly. Although food groups intake was assessed using validated FFQ, the self-reporting may always be biased. However, data on dietary intake were collected yearly to capture changes over follow-up, and a dietitian checked the FFQ with the participant to ensure that no missing data exist. Moreover, the use of FFQ does not capture sufficient details on how the food is prepared and consumed. Despite using portion sizes, residual and unmeasured variation in portion sizes might influence the associations. Due to the high number of food groups studied chance finding cannot be excluded, and as in any observational study the causality cannot be inferred.

Besides the long-term prospective design, direct measurements of anthropometry, and assessment of concurrent changes in diet with anthropometry evaluated along the same timespan, strengths of this study also include large sample size, wide spectrum of foods comprised only healthy or unhealthy items, use of standardized protocols and validated tools for anthropometry and dietary measurements, assessment of habitual dietary pattern (without energy-restrictions, supplementation or physical activity program), control for covariables, and inclusion of LOCF method. In contrary to prior observational studies [3–5], study sample included elderly participants with a great prevalence of obesity and health risk, who comprise increasing component in our societies.

In conclusion, this prospective study on concurrent changes in diet and anthropometry performed among elderly subjects at high CVD risk, revealed that rather than focusing on total calories amount, modifications in the consumption of specific food groups have a potential to prevent overall and abdominal obesity. Future studies with more precise imaging techniques are warranted to confirm findings on WC. Finally, the effect of dietary patterns rather than individual food groups, in combination with other lifestyle determinants should be studied, in order to better extrapolate these findings into public health recommendations.

## Supplementary information

**Additional file 1: Figure S1.** Flow chart summarizing selection of PREDIMED participants for the present study. **Figure S2.** Directed acyclic graph (DAG). **Table S1.** Characterization of food items belonging to each food group. **Table S2.** Correlations matrix between food groups (serving/d) within each global dietary score (GDS). **Table S3.** Sensitivity analyses: Association of yearly changes in food groups intake with concurrent changes in waist circumference over 5-year of follow-up by sex. **Table S4.** Sensitivity analysis: association of changes in global dietary score (GDS) with body weight and waist circumference changes over 5-year of follow-up. **Table S5.** Sensitivity analysis: association of changes in food groups intake for which associations with body weight (A) and waist circumference (B) changes over 5-year of follow-up were statistically significant.

## Data Availability

The datasets generated and/or analyzed during the current study are not publicly available due to the PREDIMED confidentiality policies.

## Abbreviations

BMI: Body mass index
CVD: Cardiovascular disease
DAG: Directed acyclic graph
EVOO: Extra-virgin olive oil
FFQ: Food Frequency Questionnaire
GDS: Global dietary score
GDS-WC: Global dietary score for waist circumference
GDS-Wt: Global dietary score for body weight
GEE: Generalized estimating equation
GI: Glycemic index
LOCF: Last observation carried-forward
MedDiet: Mediterranean diet
PREDIMED: Prevención con Dieta Mediterránea
SFA: Saturated fatty acid
SSB: Sugar-sweetened beverages
T2D: Type 2 diabetes
WC: Waist circumference
