# Supplementary material for: Longitudinal association of changes in diet with changes in body weight and waist circumference in subjects at high cardiovascular risk: the PREDIMED trial
Source: Int J Behav Nutr Phys Act. 2019 Dec 27;16:139. doi: 10.1186/s12966-019-0893-3 (PMC6935084; doi:10.1186/s12966-019-0893-3)
Supplement: Supplementary file 1 — Additional file 1: Figure S1. Flow chart summarizing selection of PREDIMED participants for the present study. Figure S2. Directed acyclic graph (DAG). Table S1. Characterization of food items belonging to each food group. Table S2. Correlations matrix between food groups (serving/d) within each global dietary score (GDS). Table S3. Sensitivity analyses: Association of yearly changes in food groups intake with concurrent changes in waist circumference over 5-year of follow-up by sex. Table S4. Sensitivity analysis: association of changes in global dietary score (GDS) with body weight and waist circumference changes over 5-year of follow-up. Table S5. Sensitivity analysis: association of changes in food groups intake for which associations with body weight (A) and waist circumference (B) changes over 5-year of follow-up were statistically significant. [file 12966_2019_893_MOESM1_ESM.docx]

**Additional file 1:** **Figure S1.**

**Flow chart summarizing selection of PREDIMED participants for the present study.**

**Additional file 1:** **Figure S2.**


 **Directed acyclic graph (DAG).** The total unconfounded association of long-term change in dietary intake (serving/d) with concurrent body weight (kg) and WC (cm) changes, drawn and analyzed using DAGitty ([www.dagitty.net](http://www.dagitty.net)). The minimally sufficient adjustment set (total effect) for both outcomes was composed of the following variables time, sex, age, center, intervention group (both intervention arms combined or controls), baseline BMI/WC and educational level (higher education/technician, secondary education, primary education/illiterate or missing data), as well as yearly measured changes in smoking status (never, current or former) and physical activity (METs. min/d) (continuous variable).

*Abbreviations: BMI – body mass index; WC – waist circumference.*

**Additional file 1: Table S1. Characterization of food items belonging to each food group.**

| **Food group** | **Food item** |
| --- | --- |
| Whole-fat milk | Whole milk |
| Low-fat milk | Skimmed or semi-skimmed milk |
| Whole-fat yogurt | Whole yogurts |
| Low-fat yogurt | Skimmed yogurts |
| Cheese | Cured or semi-cured cheese, white or cottage cheese |
| Eggs | Eggs |
| White meat | Turkey chicken (with/without skin), rabbit |
| Red meat | Veal, pork, lamb |
| Processed meat | Ham, serrano ham, sausages, hamburgers, bacon, pate |
| Fish and seafood | Lean fish, oily fish, salted fish, oysters, clams, mussels, squid, octopus, baby squid, cuttlefish, crustaceans (prawns, scampi, nephrops) |
| Fruits | Orange, grapefruit, mandarin, banana, apple, pear, strawberries, cherries, plums, peach, apricot, nectarine, watermelon, melon, kiwi, grapes |
| Vegetables | Chard, spinach, cabbage, cauliflower, broccoli, lettuce, endives, endive, raw tomato, carrot, pumpkin, green beans, eggplant, zucchini, cucumbers, peppers, asparagus, artichokes, leeks, thistle, celery, onion, garlic, mushrooms, chanterelles, champignon |
| Potatoes | Roasted or cooked potatoes, homemade french fries |
| Legumes | Lentils, beans, chickpeas, peas, beans |
| Nuts | Almonds, peanuts, hazelnuts, pistachios, pine nuts, nuts |
| Wholegrain bread | Bread made with unrefined flour, rye bread |
| White bread | Bread made with refined flour, sliced bread |
| Breakfast cereals | Whole grains (muesli, flakes, oatmeal, all-bran) |
| Refined grains | Pasta, noodles, macaroni, spaghetti, white rice |
| Olive oil | Olive oil |
| EVOO | Extra virgin olive oil |
| Other vegetable oils | Oils from olive-pomace, corn, sunflower, soybean, mixture of the above |
| Margarine | All type of margarines |
| Butter | All types of butters |
| Snacks, fast foods and pre-prepared dishes | Croquettes, fritters (*esp*. buñuelos), dumplings (*esp.* empanadillas), pre-cooked, instant soups and creams, pizza, worms, popcorn, corn, mustard, commercial mayonnaise, commercial fried tomato sauce, ketchup, commercial french fries |
| Sweets | Breakfast cookies, integral or fiber biscuits, cookies with chocolate, home-made pastries and biscuits, croissant, regional pastry (*esp*. ensaimada), commercial pastries and biscuits, donuts, muffins, cakes, chocolates and candies, cocoa powder, fat cakes, marzipan, nougat, jam, sugar, honey |
| SSB | Soft beverages with sugar (drinks with cola, lemonades, tonic, fruit juices (bottled or canned)) |
| ASB | Low-calorie soft beverages, light beverages |
| Natural juices | Natural fruit juices |
| Coffee/tea | Coffee (classic, decaffeinated), black tea |
| Alcoholic beverages | Wines (rosé, muscatel, young red, year old red, white, cava) beers, liqueurs (including anise-flavored), distilled (whiskey, vodka, gin, cognac) |

Abbreviations: ASB – artificially-sweetened beverages; EVOO – extra virgin olive oil; SSB – sugar-sweetened beverages.

| **GDS-Wt** | Red  meat | Processed  meat | Refined  grains | White  bread | Potatoes | Low-fat  yogurt | Low-fat  milk | Sweets | Alcoholic beverages |
| --- | --- | --- | --- | --- | --- | --- | --- | --- | --- |
| Red meat | 1.000 |  |  |  |  |  |  |  |  |
| Processed meat | 0.128 | 1.000 |  |  |  |  |  |  |  |
| Refined grains | 0.061 | 0.096 | 1.000 |  |  |  |  |  |  |
| White bread | 0.040 | 0.056 | 0.085 | 1.000 |  |  |  |  |  |
| Potatoes | 0.070 | 0.083 | 0.127 | 0.071 | 1.000 |  |  |  |  |
| Low-fat yogurt | -0.032 | -0.028 | -0.021 | -0.026 | -0.011 | 1.000 |  |  |  |
| Low-fat milk |  | 0.014 | 0.033 |  | 0.016 | 0.031 | 1.000 |  |  |
| Sweets | 0.069 | 0.081 | 0.051 | 0.043 | 0.048 | -0.017 | 0.037 | 1.000 |  |
| Alcoholic beverages | 0.032 | 0.062 | 0.024 | 0.034 | 0.027 | -0.029 | -0.015 | 0.045 | 1.000 |

**Additional file 1:** **Table S2.** Correlations matrix between yearly change in food groups intake (serving/d) within each global dietary score (GDS).

| **GDS-WC** | Processed meat | Vegetables | Nuts | Snacks, fast-foods and pre-prepared dishes | Sweets | Alcoholic beverages |
| --- | --- | --- | --- | --- | --- | --- |
| Processed meat | 1.000 |  |  |  |  |  |
| Vegetables | 0.068 | 1.000 |  |  |  |  |
| Nuts | 0.019 | 0.122 | 1.000 |  |  |  |
| Snacks, fast-foods and pre-prepared dishes | 0.100 | 0.069 | 0.033 | 1.000 |  |  |
| Sweets | 0.081 | 0.020 |  | 0.082 | 1.000 |  |
| Alcoholic beverages | 0.062 | 0.032 | 0.020 | 0.067 | 0.045 | 1.000 |

Values shown are those pairwise Pearson correlation coefficients (r) that were statistically significant (p < 0.05; Pearson correlation).

The GDSs were constructed at baseline and yearly during follow-up considering foods that were significantly associated with weight and waist circumference changes (*p*<0.05; generalized estimating equation). Both GDSs were classified into quintiles and for each of them different values have been assigned depending whether the association with the anthropometry was negative (1 to 5) or positive (5 to 1). After summing all of these values the final score ranged from 9 to 45 points for body weight and from 6 to 30 points for WC; a higher score indicated a dietary pattern associated with less weight or WC gain. For the analyses GDSs were normalized into z-scores.

*Abbreviations: GDS-Wt – global dietary score for body weight; GDS-WC – global dietary score for waist circumference.*

**Additional file 1:** **Table S3. Sensitivity analyses: Association of yearly changes in food groups intake with concurrent changes in waist circumference over 5-year**

|  | **MEN** | | **WOMEN** | |
| --- | --- | --- | --- | --- |
| **Food group (serving/d)** | **β (95% CI)** | ***p-value*** | **β (95% CI)** | ***p-value*** |
| Alcoholic beverages | 0.13 (0.02, 0.23) | *0.021* | 0.17 (-0.12, 0.46) | *0.253* |
| ASB | 0.25 (-0.04, 0.53) | *0.096* | 0.00 (-0.37, 0.37) | *0.993* |
| Breakfast cereals | 0.18 (-0.19, 0.56) | *0.339* | -0.03 (-0.46, 0.40) | *0.889* |
| Butter | -0.38 (-0.97, 0.22) | *0.214* | 0.06 (-0.69, 0.82) | *0.866* |
| Cheese | 0.02 (-0.20, 0.25) | *0.848* | 0.10 (-0.19, 0.38) | *0.510* |
| Coffee/tea | 0.13 (0.00, 0.25) | *0.042* | 0.04 (-0.10, 0.18) | *0.566* |
| Eggs | 0.27 (-0.34, 0.88) | *0.386* | -0.68 (-1.49, 0.12) | *0.096* |
| EVOO | -0.01 (-0.07, 0.04) | *0.650* | -0.06 (-0.12, 0.00) | *0.039* |
| Fish and seafood | -0.22 (-0.51, 0.08) | *0.150* | 0.10 (-0.25, 0.45) | *0.577* |
| Fruits | -0.02 (-0.15, 0.11) | *0.761* | -0.08 (-0.22, 0.06) | *0.288* |
| Legumes | 0.00 (-1.40, 1.40) | *0.999* | -0.79 (-2.45, 0.88) | *0.355* |
| Low-fat milk | -0.11 (-0.26, 0.03) | *0.136* | -0.01 (-0.16, 0.14) | *0.935* |
| Low-fat yogurt | -0.09 (-0.49, 0.30) | *0.640* | 0.09 (-0.25, 0.42) | *0.606* |
| Margarine | 0.25 (-0.30, 0.80) | *0.373* | -0.31 (-0.86, 0.24) | *0.267* |
| Natural juices | -0.10 (-0.48, 0.28) | *0.605* | -0.28 (-0.76, 0.21) | *0.259* |
| Nuts | 0.11 (-0.08, 0.30) | *0.269* | -0.41 (-0.65, -0.17) | *0.001* |
| Olive oil | 0.02 (-0.05, 0.09) | *0.577* | 0.03 (-0.04, 0.11) | *0.419* |
| Other vegetable oils | 0.09 (-0.10, 0.28) | *0.345* | 0.16 (-0.06, 0.38) | *0.155* |
| Potatoes | -0.22 (-0.60, 0.16) | *0.256* | 0.40 (-0.08, 0.87) | *0.102* |
| Processed meat | 0.05 (-0.13, 0.24) | *0.559* | 0.31 (0.05, 0.56) | *0.017* |
| Red meat | 0.12 (-0.30, 0.55) | *0.564* | 0.57 (-0.03, 1.18) | *0.062* |
| Refined grains | 0.41 (-0.23, 1.05) | *0.207* | 0.27 (-0.37, 0.90) | *0.409* |
| Snacks, fast-foods and pre-prepared dishes | 0.18 (-0.12, 0.48) | *0.238* | 0.36 (0.04, 0.69) | *0.027* |
| SSB | 0.00 (-0.32, 0.32) | *0.982* | 0.14 (-0.18, 0.46) | *0.395* |
| Sweets | 0.07 (0.01, 0.12) | *0.013* | 0.10 (0.03, 0.16) | *0.003* |
| Vegetables | -0.16 (-0.36, 0.04) | *0.111* | -0.28 (-0.53, -0.03) | *0.028* |
| White bread | 0.00 (-0.11, 0.10) | *0.951* | 0.08 (-0.05, 0.21) | *0.242* |
| White meat | -0.27 (-0.75, 0.22) | *0.280* | -0.16 (-0.74, 0.42) | *0.588* |
| Whole bread | -0.12 (-0.29, 0.04) | *0.135* | 0.01 (-0.19, 0.21) | *0.911* |
| Whole-fat milk | -0.04 (-0.29, 0.21) | *0.751* | 0.22 (-0.10, 0.54) | *0.178* |
| Whole-fat yogurt | -0.04 (-0.46, 0.38) | *0.854* | 0.16 (-0.29, 0.61) | *0.490* |

**of follow-up by sex.**

β (95% CI) represents the yearly change in waist circumference (cm) associated with increased/decreased consumption of particular food groups (serving/d) in men and women. The principal model used for generalized estimating equation analysis was adjusted for time, center, intervention group, age, baseline waist circumference and educational level, as well as yearly measured changes in smoking status and physical activity.

*Abbreviations: ASB – artificially-sweetened beverages; EVOO – extra virgin olive oil; SSB – sugar-sweetened beverages*

**Additional file 1:** **Table S4. Sensitivity analysis: association of changes in global dietary score (GDS) with body weight and waist circumference changes over 5-year of follow-up.**

|  | **Body weight change (kg)** | | **Waist circumference change (cm)** | |
| --- | --- | --- | --- | --- |
| **GDS (per 1 SD)** | β (95% CI) | *p*-value | β (95% CI) | *p*-value |
| Completers-only^1^ | -0.17 (-0.22, -0.12) | <0.0001 | -0.29 (-0.36, -0.21) | <0.0001 |
| Change in energy intake^2^ | -0.13 (-0.18, -0.08) | <0.0001 | -0.27 (-0.35, -0.19) | <0.0001 |
| Excluding patients who died^3^ | -0.16 (-0.21, -0.12) | <0.0001 | -0.28 (-0.36, -0.20) | <0.0001 |
| Excluding 2^nd^ household members^4^ | -0.17 (-0.22, -0.12) | <0.0001 | -0.27 (-0.35, -0.19) | <0.0001 |
| Excluding site D^5^ | -0.17 (-0.22, -0.12) | <0.0001 | -0.26 (-0.34, -0.18) | <0.0001 |
| Changes in adherence to MedDiet^6^ | -0.16 (-0.21, -0.12) | <0.0001 | -0.26 (-0.34, -0.18) | <0.0001 |

β (95% CI) represents the yearly change in body weight or waist circumference associated with yearly increments of each GDS. The principal model used for generalized estimating equation analysis was adjusted for time, sex, center, intervention group, age, baseline BMI/WC (depending on outcome) and educational level, as well as yearly measured changes in smoking status and physical activity.

^1^Calculated with data of completers-only, without the LOCF imputation of missing data.

^2^Additionally adjusted for yearly measured changes in total energy intake.

^3^Calculated excluding patients who died for any cause (n=348).

^4^Calculated excluding participants who were not individually allocated but randomized by small cluster (2^nd^ household members were enrolled to the same intervention group as a previously enrolled participants) (n=407).

^5^Calculated excluding participants who were not individually allocated but randomized by small clusters (clinics) at one of eleven recruitment sites (site D) (n=527).

^6^Additionally adjusted for yearly measured changes in adherence to MedDiet (14-points score).

**Additional file 1:** **Table S5. Sensitivity analysis: association of changes in food groups intake for which associations with body weight (A) and waist circumference (B) changes over 5-year of follow-up were statistically significant.**

|  |  | Alcoholic beverages | Processed meat | Red meat | Potatoes | White bread | Refined grains | Low-fat  milk | Low-fat  yogurt |
| --- | --- | --- | --- | --- | --- | --- | --- | --- | --- |
| Completers-only^1^ | β  (95% CI) | 0.15  (0.08, 0.22) | 0.14  (0.04, 0.23) | 0.29  (0.07, 0.52) | 0.20  (0.04, 0.36) | 0.06  (0.01, 0.10) | 0.26  (0.01, 0.51) | -0.07  (-0.13, -0.01) | -0.14  (-0.29, 0.00) |
|  | *p-value* | ***<0.0001*** | ***0.006*** | ***0.011*** | ***0.014*** | ***0.016*** | ***0.038*** | ***0.017*** | *0.056* |
| Change in energy intake^2^ | β  (95% CI) | 0.15  (0.07, 0.23) | 0.08  (-0.03, 0.18) | 0.10  (-0.13, 0.34) | 0.10  (-0.08, 0.27) | 0.01  (-0.05, 0.07) | 0.12  (-0.11, 0.36) | -0.09  (-0.15, -0.03) | -0.19  (-0.33, -0.04) |
|  | *p-value* | ***<0.0001*** | *0.141* | *0.378* | *0.271* | *0.698* | *0.310* | ***0.004*** | ***0.011*** |
| Excluding patients who died^3^ | β  (95% CI) | 0.19  (0.12, 0.27) | 0.16  (0.05, 0.26) | 0.26  (0.03, 0.49) | 0.23  (0.06, 0.40) | 0.06  (0.01, 0.12) | 0.34  (0.11, 0.58) | -0.06  (-0.12, 0.00) | -0.19  (-0.34, -0.04) |
|  | *p-value* | ***<0.0001*** | ***0.003*** | ***0.025*** | ***0.007*** | ***0.015*** | ***0.004*** | *0.062* | ***0.011*** |
| Excluding 2^nd^ household members^4^ | β  (95% CI) | 0.17  (0.10, 0.25) | 0.16  (0.06, 0.27) | 0.22  (-0.01, 0.45) | 0.26  (0.09, 0.44) | 0.07  (0.02, 0.12) | 0.35  (0.11, 0.59) | -0.06  (-0.12, 0.00) | -0.18  (-0.32, -0.03) |
|  | *p-value* | ***<0.0001*** | ***0.003*** | *0.062* | ***0.003*** | ***0.008*** | ***0.004*** | *0.066* | ***0.016*** |
| Excluding site D^5^ | β  (95% CI) | 0.20  (0.13, 0.28) | 0.15  (0.05, 0.26) | 0.23  (0.00, 0.46) | 0.24  (0.06, 0.42) | 0.06  (0.01, 0.12) | 0.30  (0.06, 0.54) | -0.06  (-0.13, 0.00) | -0.18  (-0.33, -0.03) |
|  | *p-value* | ***<0.0001*** | ***0.005*** | *0.056* | ***0.008*** | ***0.017*** | ***0.015*** | *0.050* | ***0.021*** |
| Change in adherence to MedDiet^6^ | β  (95% CI) | 0.18  (0.11, 0.26) | 0.15  (0.05, 0.26) | 0.26  (0.04, 0.48) | 0.23  (0.06, 0.40) | 0.07  (0.02, 0.12) | 0.32  (0.09, 0.54) | -0.06  (-0.13, 0.00) | -0.18  (-0.33, -0.04) |
|  | *p-value* | ***<0.0001*** | ***0.003*** | ***0.020*** | ***0.008*** | ***0.009*** | ***0.006*** | ***0.036*** | ***0.012*** |

**A. Body weight change (kg)**

1. **Waist circumference change (cm)**

|  |  | Alcoholic beverages | Processed meat | Snacks, fast-foods and pre-prepared dishes | Sweets | Vegetables | Nuts |
| --- | --- | --- | --- | --- | --- | --- | --- |
| Completers-only^1^ | β  (95% CI) | 0.16  (0.07, 0.26) | 0.25  (0.11, 0.40) | 0.35  (0.14, 0.56) | 0.10  (0.06, 0.14) | -0.31  (-0.45, -0.16) | -0.14  (-0.29, 0.01) |
|  | *p-value* | ***0.001*** | ***0.001*** | ***0.001*** | ***<0.0001*** | ***<0.0001*** | ***0.072*** |
| Change in energy intake^2^ | β  (95% CI) | 0.12  (0.02, 0.23) | 0.16  (0.01, 0.32) | 0.26  (0.04, 0.48) | 0.08  (0.04, 0.13) | -0.27  (-0.44, -0.10) | -0.21  (-0.38, -0.05) |
|  | *p-value* | ***0.019*** | ***0.042*** | ***0.021*** | ***<0.0001*** | ***0.002*** | ***0.011*** |
| Excluding patients who died^3^ | β  (95% CI) | 0.15  (0.04, 0.25) | 0.18  (0.02, 0.34) | 0.29  (0.06, 0.52) | 0.08  (0.04, 0.13) | -0.22  (-0.38, -0.05) | -0.15  (-0.30, 0.01) |
|  | *p-value* | ***0.006*** | ***0.024*** | ***0.013*** | ***<0.0001*** | ***0.010*** | *0.071* |
| Excluding 2^nd^ household members^4^ | β  (95% CI) | 0.13  (0.02, 0.23) | 0.15  (-0.01, 0.30) | 0.33  (0.11, 0.56) | 0.08  (0.03, 0.12) | -0.21  (-0.38, -0.04) | -0.16  (-0.32, 0.00) |
|  | *p-value* | ***0.020*** | *0.068* | ***0.004*** | ***<0.0001*** | ***0.014*** | *0.054* |
| Excluding site D^5^ | β  (95% CI) | 0.14  (0.03, 0.24) | 0.15  (-0.01, 0.31) | 0.31  (0.08, 0.54) | 0.08  (0.04, 0.12) | -0.23  (-0.40, -0.06) | -0.16  (-0.32, 0.00) |
|  | *p-value* | ***0.012*** | *0.074* | ***0.009*** | ***<0.0001*** | ***0.007*** | ***0.049*** |
| Change in adherence to MedDiet^6^ | β  (95% CI) | 0.14  (0.04, 0.24) | 0.16  (0.01, 0.31) | 0.28  (0.05, 0.50) | 0.08  (0.04, 0.12) | -0.20  (-0.37, -0.04) | -0.13  (-0.29, 0.03) |
|  | *p-value* | **0.007** | ***0.042*** | **0.015** | ***<0.0001*** | ***0.017*** | *0.118* |

β (95% CI) represents the yearly change in body weight (kg) or waist circumference (cm) associated with increased/decreased consumption of particular food groups (serving/d). The principal model used for generalized estimating equation analysis was adjusted for time, sex, center, intervention group, age, baseline BMI/WC (depending on outcome) and educational level, as well as yearly measured changes in smoking status and physical activity.

^1^Calculated with data of completers-only, without the LOCF imputation of missing data.

^2^Additionally adjusted for yearly measured changes in total energy intake.

^3^Calculated excluding patients who died for any cause (n=348).

^4^Calculated excluding participants who were not individually allocated but randomized by small cluster (2^nd^ household members were enrolled to the same intervention group as a previously enrolled participants) (n=407).

^5^Calculated excluding participants who were not individually allocated but randomized by small clusters (clinics) at one of eleven recruitment sites (site D) (n=527).

^6^Additionally adjusted for yearly measured changes in adherence to MedDiet (14-points score).
